# Smoking-attributable ischemic heart disease burden in China from 1990 to 2021 and projections to 2036: A retrospective analysis and forecasting study

**DOI:** 10.18332/tid/217625

**Published:** 2026-03-21

**Authors:** Dingwen Xu, Zhe Yang, Weijuan Yao, Xifu Wang

**Affiliations:** 1 Department of Clinic, School of Medicine, Yangzhou Polytechnic University, Yangzhou, China; 2 Department of Emergency, Beijing Anzhen Hospital, Capital Medical University, Beijing, China; 3 Department of Orthopedics, Shanxi Provincial People’s Hospital, Taiyuan, China; 4 Hemorheology Center, Department of Physiology and Pathophysiology, School of Basic Medical Sciences, Peking University Health Science Center, Beijing, China

**Keywords:** smoking-related ischemic heart disease, China, GBD, CHARLS

## Abstract

**INTRODUCTION:**

Ischemic heart disease (IHD) remains a leading global health challenge, with smoking identified as a key modifiable risk factor. China, with its high smoking prevalence and aging population, faces a growing burden of smoking-related IHD. This study evaluates the disease burden of smoking-related IHD in China from 1990 to 2021 and projects future trends to inform targeted interventions.

**METHODS:**

Using data from the Global Burden of Disease (GBD) 2021 (1990–2021) and the cross-sectional baseline survey of the China Health and Retirement Longitudinal Study (CHARLS, Wave 1, 2011–2012), we analyzed trends in mortality, disability-adjusted life years (DALYs), years lived with disability (YLDs), and years of life lost (YLLs). Joinpoint regression identified temporal trends, and an autoregressive integrated moving average (ARIMA) model forecast disease burden up to 2036. The CHARLS analysis included participants aged ≥45 years with complete smoking and health records. Multivariable logistic regression assessed the association between smoking status and heart disease likelihood in CHARLS participants.

**RESULTS:**

CHARLS data revealed that former smokers had an 84% higher likelihood of heart disease than never smokers (adjusted odds ratio, AOR=1.84; 95% CI: 1.50–2.25). GBD analysis showed that China’s age-standardized mortality rate (ASMR) for smoking-related IHD surpassed global and US levels after 2005, with males bearing a significantly higher burden than females. Joinpoint regression identified key turning points, with male ASMR rising until 2010 before declining slightly, while female indicators consistently improved. ARIMA projections suggest male ASMR will remain high (39.01; 95% UI: 22.69–55.33) by 2036, indicating persistent challenges.

**CONCLUSIONS:**

The burden of smoking-related IHD in China exceeds the global average and reveals significant gender disparities, with a worsening burden for males and improvement for females. There is a critical need for more effective smoking control measures aimed at the male population to tackle this major public health issue.

## INTRODUCTION

Ischemic heart disease (IHD) has become a major public health challenge globally in the 21st century, being the leading cause of death among non-communicable diseases (NCDs), with a continuously increasing disease burden. According to the 2019 Global Burden of Disease (GBD) data, IHD caused 9.1 million deaths worldwide, accounting for 49.2% of all cardiovascular disease (CVD)-related deaths^1^. Importantly, the increase in IHD mortality since 2000 ranks highest among all diseases and is closely associated with severe disabilities^2^. Globally, IHD results in 192 million disability-adjusted life years (DALYs) and 176 million years of life lost (YLLs) each year, imposing a heavy burden on healthcare systems and socioeconomic development^2^. In response to this severe situation, the World Health Organization (WHO) has prioritized the prevention and control of cardiovascular diseases as a global health issue, making it crucial to understand the epidemiological characteristics and distribution of risk factors for IHD to formulate effective intervention strategies^3^.

Among the numerous risk factors for IHD, smoking is recognized as one of the most harmful modifiable factors^4^. A substantial body of research has confirmed that smoking promotes the onset and progression of coronary atherosclerosis through multiple pathophysiological mechanisms, including increasing low-density lipoprotein (LDL) oxidation, impairing endothelial function, and accelerating thrombosis^5^. Prospective cohort studies have shown that smokers are more than twice as likely to develop coronary heart disease compared to non-smokers, and the mortality rate significantly increases among smokers who continue smoking after undergoing coronary intervention^6^. Despite increasing global efforts to control smoking, tobacco use remains a major risk factor for premature death and disability. In 2019, there were still 1.14 billion smokers globally, with China accounting for 30% (341 million people), and tobacco consumption reaching an average of 2150 cigarettes per person, far exceeding the global average^7^. This unique epidemiological characteristic makes China an ideal subject for studying the disease burden of smoking-related IHD.

The situation regarding IHD prevention and control in China is particularly complex. On the one hand, rapid socioeconomic development and industrialization have led to significant lifestyle changes, including a Westernized diet and reduced physical activity, which synergistically interact with traditional smoking risk factors to elevate the risk of IHD^8^. On the other hand, China is experiencing an unprecedented aging population, which further amplifies the susceptibility of the elderly to IHD and increases the disease burden^9^. Moreover, although China has implemented the ‘Healthy China 2030’ initiative, aiming to reduce the smoking rate among those aged ≥15 years to 20% by 2030, the actual effectiveness of smoking control falls significantly short of expectations^7^. As of 2022, only 20 cities nationwide had achieved comprehensive smoke-free legislation, covering <16% of the population, resulting in a continued increase in the disease burden of smoking-related IHD^7^. Therefore, this study aims to conduct a comprehensive assessment of the disease burden of smoking-related IHD in China from 1990 to 2021 using GBD 2021 data, providing a scientific basis for formulating targeted intervention measures.

## METHODS

This study constitutes a secondary analysis of existing population-level data, employing both comparative trend analysis and predictive modeling.

### CHARLS analysis

This study utilized data from the first wave of the China Health and Retirement Longitudinal Study (CHARLS) (2011–2012)^10^, with an initial sample of 17708 individuals aged ≥45 years. The CHARLS study received ethical approval from the Biomedical Ethics Review Committee at Peking University (IRB00001052-11015), and all participants provided written informed consent prior to data collection, ensuring compliance with ethical standards for human subject research. Systematic screening was conducted to ensure that all participants included in the analysis had complete data for key variables, including smoking history, current smoking status, heart disease diagnosis, age, and gender. After data cleaning, 1023 records with missing values for critical variables were excluded, accounting for 5.8% of the initial sample, resulting in 16685 participants. Furthermore, to ensure biological plausibility, we excluded an additional 107 records (0.6%) with medically implausible body mass index (BMI) values (<15 kg/m^2^ or >40 kg/m^2^) based on established clinical thresholds. The final analytic sample thus comprised 16578 participants.

All variables were clearly defined as follows: 1) Smoking status, a categorical variable based on self-reported questionnaire data, classified as never smokers (individuals who had never smoked), current smokers (those currently smoking at the time of survey), and former smokers (those who had quit smoking); 2) Heart disease diagnosis, a binary variable (yes/no) derived from self-reported questionnaire data, where participants were asked if a doctor had ever diagnosed them with any heart condition, including but not limited to ischemic heart disease (IHD). This broad category captures overall cardiac health; 3) Age, a continuous quantitative variable measured in years, based on self-reported date of birth; 4) Gender, a categorical variable (male/ female) collected via self-report; 5) Residence, a categorical variable (urban/rural) determined from self-reported location data, with rural as the reference category; 6) Hypertension, a binary variable (yes/ no) based on self-reported diagnosis or measured blood pressure criteria (systolic ≥140 mmHg or diastolic ≥90 mmHg), as per CHARLS protocol. This was considered a potential confounder due to its association with IHD; 7) Diabetes, a binary variable (yes/no) based on self-reported diagnosis or use of diabetes medication, collected via questionnaire. This was treated as a potential confounder; and 8) BMI, a continuous quantitative variable in kg/m^2^, calculated from measured height and weight during CHARLS assessments. Values were categorized as implausible if outside the range of 15–40 kg/m^2^ for quality control.

Data collection methods primarily involved face-to-face interviews and standardized questionnaires in CHARLS, which may include self-reported elements for variables like heart disease, hypertension, and diabetes. All variables except age and BMI were treated as categorical or binary in analyses, and covariates such as age, gender, residence, hypertension, diabetes, and BMI were included as potential confounders in the regression model due to their known associations with heart disease outcomes.

It is important to note that the CHARLS survey recorded heart disease as a broad category, which encompasses various cardiac conditions including but not limited to ischemic heart disease (IHD). While this lacks the specificity of the IHD-specific data from the GBD study, it serves as a robust population-level indicator for assessing the association between smoking and overall cardiac health, which is strongly driven by IHD.

### Data collection from GBD

This study is based on the Global Burden of Disease (GBD) 2021 database (1990–2021)^11^, systematically extracting data on the disease burden of smoking-related IHD in the Chinese population from 1990 to 2021. The IHD case definition in the GBD study is based on the International Classification of Diseases (ICD) codes, primarily ICD-10 codes I20-I25. The core indicators include Deaths, DALYs (disability-adjusted life years), YLDs (years lived with disability), and YLLs (years of life lost), with their respective numbers and age-standardized rates, referred to as ASMR (age-standardized mortality rate), ASDR (age-standardized disability rate), ASYLD (age-standardized years lived with disability), and ASYLL (age-standardized years of life lost), where DALYs = YLLs + YLDs^12^. All indicators provide point estimates and 95% uncertainty intervals (UI), derived from the unique 1000 ordered estimates method used in GBD research, ensuring data reliability^13^. Additionally, the estimated annual percentage change (EAPC) reflects the overall changes from 1990 to 2021, calculated using a logarithmic linear regression model [ln(rate) = α + β × year + ε], where α represents the intercept of the regression line, β is the slope coefficient indicating the average annual change in the log-transformed rate, and ε denotes the error term accounting for residual variability. The EAPC value is derived as [exp(β) - 1] × 100%. An EAPC with a 95% confidence interval (CI) lower limit greater than 0 indicates an upward trend, while an upper limit less than 0 indicates a downward trend; otherwise, the trend is considered stable^14^. The study specifically focuses on differences between genders, conducting independent analyses for male and female data. During data processing, strict quality control measures were implemented, including outlier detection using the interquartile range (IQR) method to identify and address extreme values, missing value handling through multiple imputation techniques to preserve data integrity, and consistency verification by cross-referencing with external sources to ensure data reliability, thereby ensuring the accuracy of the analysis results.

The 95% UIs associated with the GBD estimates are designed to capture the total uncertainty arising from all steps of the modeling process, including data sampling error, model selection, and parameter estimation. In contrast, the 95% CIs cited for metrics like the EAPC quantify the uncertainty specific to the statistical model’s parameters based on the variability of the input data.

It is noteworthy that all data used in this study are publicly available aggregated data, and do not involve any individual information, thus exempting the study from requiring specific ethical committee review and approval.

### Joinpoint regression analysis

To analyze the temporal trends in disease burden, this study employed the Joinpoint regression method. Utilizing the Joinpoint software (version 4.9.1.0) developed by the National Cancer Institute of China, a model was established to calculate the average annual percentage change (AAPC) to quantify the magnitude of overall trend changes. The model uses segmented linear regression to objectively identify time points where significant changes in disease rates occur. The standard error (SE) is calculated using the formula (upper UI - lower UI)/(2×1.96), with a statistical significance level set at p<0.05 ^15^. This method effectively avoids the subjectivity limitations that may arise from traditional linear trend analyses, providing a reliable basis for long-term trend analysis of disease burden.

### ARIMA forecasting analysis

For predictive analysis, this study constructed an autoregressive integrated moving average (ARIMA) model to forecast future changes in disease burden. The modeling process includes key steps such as stationarity testing, parameter selection, and model validation. After ensuring data stationarity through differencing, the maximum likelihood method was used to estimate model parameters, with the Akaike information criterion (AIC) employed for model selection. The predictive capability of the model was assessed using metrics such as mean absolute percentage error (MAPE) and root mean square error (RMSE)^16^. The final predictions include point estimates and 95% confidence intervals for the years 2022–2036. This forecast is based on the ARIMA model, which rests on several key assumptions, including the stability of smoking prevalence, the absence of significant changes in existing tobacco control policies, and the continuation of current demographic trends. The potential impacts of unforeseen public health events or policy interventions are not accounted for in this analysis.

### Statistical analysis

Descriptive statistics of CHARLS analysis, including means and percentages for continuous and categorical variables, respectively, were calculated for the entire sample and by smoking status groups to characterize the population. For example, age was summarized as mean ± standard deviation, and categorical variables like gender, residence, hypertension, and diabetes were reported as frequencies and percentages. For statistical analysis, Pearson’s chi-squared test was first employed to compare the prevalence of heart disease across the three groups. To further control for potential confounders, a multivariable logistic regression model was constructed, with heart disease diagnosis as the dependent variable and smoking status as the primary independent variable. Covariates included age, gender (male as reference), residence (rural as reference), hypertension, diabetes, and BMI. Regression results are presented as odds ratios (ORs) with 95% confidence intervals. All statistical analyses were performed using R software (version 4.3.1), with a significance level set at α=0.05.

## RESULTS

### Smoking is closely associated with heart disease in the Chinese population: evidence from CHARLS data

As shown in the Table 1, the study included a total of 16578 participants from the CHARLS baseline survey, with smoking status categorized as never smokers (n=10407), current smokers (4786), and former smokers (1385). Baseline characteristics indicated significant differences among the three groups in terms of demographic and health profiles (all p<0.05), with former smokers having the highest mean age (62.6 ± 10.2 years) and the highest prevalence of hypertension (34.1%). Crude analysis demonstrated a distinctive distribution pattern in heart disease prevalence: former smokers (18.1%) > never smokers (12.7%) > current smokers (9.5%) (χ^2^=81.88, p<0.001) (Table 1). Univariate logistic regression analysis further confirmed these patterns, revealing that former smokers had a significantly higher crude odds of heart disease compared to never smokers (OR=1.52; 95% CI: 1.30–1.76, p<0.001), while current smokers had a significantly lower crude odds (OR=0.72; 95% CI: 0.64–0.81, p<0.001). After adjusting for confounders such as age, sex, residence, hypertension, diabetes, and BMI, former smokers exhibited a markedly increased likelihood of heart disease compared to never smokers (AOR=1.84; 95% CI: 1.50–2.25, p<0.001), whereas the likelihood among current smokers (AOR=1.15; 95% CI: 0.97–1.37, p=0.096) did not reach statistical significance.

**Table 1:** Association between smoking status and heart disease risk in the Chinese population, CHARLS 2011–2012

*Variable*	*Never smoker* *(N=10407)* *%*	*Current smoker* *(N=4786)* *%*	*Former smoker* *(N=1385)* *%*	*p*	*AOR (95% CI)*
**Demographics**					
Age (years), mean ± SD	58.3 ± 10.4	59.0 ± 9.3	62.6 ± 10.2	<0.001	
Male	20.8	89.5	87.9	<0.001	
Rural residence	58.3	63.3	57.0	<0.001	
**Health status**					
Hypertension	26.8	21.9	34.1	<0.001	
Diabetes	6.6	4.2	8.8	<0.001	
BMI (kg/m²), mean ± SD	23.9 ± 3.7	22.5 ± 3.3	23.7 ± 3.6	<0.001	
**Heart disease**					
Cases, n	1326	456	251		
Crude prevalence	12.7	9.5	18.1	<0.001	
**Smoking**					
Never (ref.)					1.00
Adjusted current vs never				0.096	1.15 (0.97–1.37)
Adjusted former vs never				<0.001	1.84 (1.50–2.25)

AOR: adjusted odds ratio; adjusted for age, sex, residence, hypertension, diabetes, and BMI.

### Trends in smoking-related IHD burden in China, the United States, and globally

**Figure 1:**
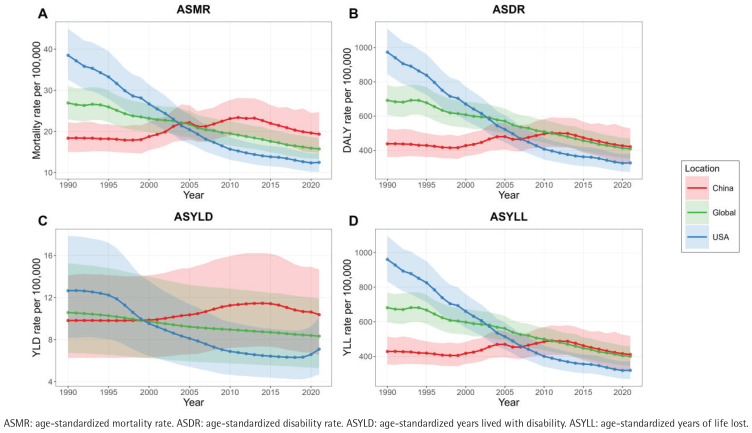
China’s rising and higher burden of smoking-related IHD compared to the United States and Global levels, 2000–2021: A) ASMR, B) ASDR, C) ASYLD, D) ASYLL. The shaded areas around trend lines represent the 95% UI for age-standardized rates

It is evident that prior to 2005, China’s ASMR was consistently lower than both the global level and that of the United States. However, after 2005, China’s ASMR began to rise and has remained higher than both the global average and the United States, following the same trends through 2021. In terms of ASDR, China remained below the global average until 2011, after which it has been slightly above the global level. Of significance, since 2007, China’s ASDR has significantly exceeded that of the United States. The ASYLD has consistently been higher than both the global level and the United States since 2000. Additionally, ASYLL has been higher than the United States since 2007 and has remained slightly above the global average since 2012 (Figure 1).

### Age and gender disparities in smoking-related IHD burden in China (2021)

**Figure 2:**
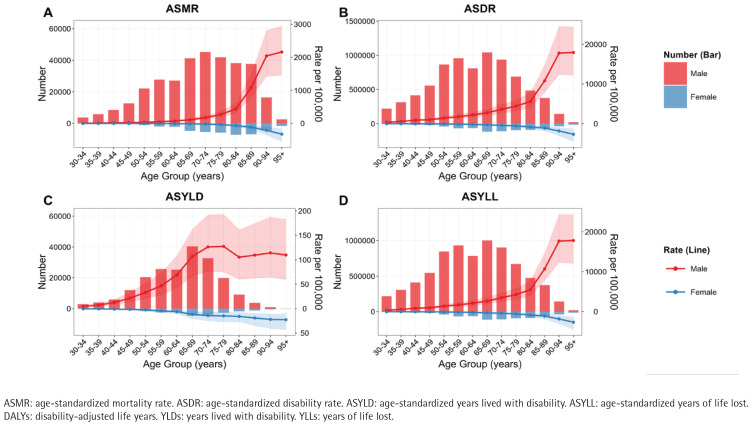
Substantially higher burden of smoking-related IHD among males and older adults in China, 2021: A) Deaths and ASMR, B) DALYs and ASDR, C) YLDs and ASYLD, D) YLLs and ASYLL. The shaded areas around trend lines represent the 95% UI for age-standardized rates

Most deaths occur in the age group of 60–89 years, while the peak ASMR is observed in the age group of ≥90 years (Figure 2A). The DALYs are mainly concentrated in the age group of 50–79 years, with the highest ASDR also occurring in those aged ≥90 years (Figure 2B). The YLDs are predominantly found in the 50–79 years group, while the ASYLD remains elevated after the age of 70 years (Figure 2C). The YLLs are primarily concentrated in the 50–79 years group, with the peak ASYLL occurring in those aged ≥90 years (Figure 2D).

Overall, the disease burden is significantly higher in males compared to females. Specifically, the number of deaths for males is 331587 (95% UI: 251500–429418) versus 42236 (95% UI: 27752–60634) for females, resulting in an ASMR of 39.9 (95% UI: 29.92–52.05) per 100000 for males compared to 4.08 (95% UI: 2.63–5.9) per 100000 for females (Figure 2A, Table 2). The DALYs for males stand at 7834073 (95% UI: 5977996–10052681) versus 759830 (95% UI: 52396–1050934) for females, with an ASDR of 820.81 (95% UI: 630.03–1045.28) per 100000 for males and 70.48 (95% UI: 48.36–98.16) per 100000 for females (Figure 2B, Table 2). The YLDs for males are 203043 (95% UI: 127224–288389) compared to 22470 (95% UI: 13319–33049) for females, with an ASYLD of 19.28 (95% UI: 12–27.29) per 100000 for males versus 2.01 (95% UI: 1.21–2.96) per 100000 for females (Figure 2C, Table 2). The number of YLLs for males is 7631031 (95% UI: 5786889–9859840) compared to 737361 (505820–1030964) for females, with an ASYLL of 801.52 (95% UI: 611.2–1023.37) per 100000 for males versus 68.47 (95% UI: 46.77–96.1) per 100000 for females (Figure 2D, Table 2).

**Table 2:** All-age cases of smoking-related ischemic heart disease (IHD) in China in 1990 and 2021, and ASMR, ASDR, ASYLD, and ASYLL (GBD 1990–2021)

		*Both*	*Male*	*Female*
**1990**	Deaths (95% UI)	132822 (109924–160264)	111517 (89317–135453)	21305 (16139–27855)
	ASMR per 10^5^ (95% UI)	18.37 (15.04–22.22)	33.8 (26.91–41.4)	6.45 (4.71–8.74)
	DALYs (95% UI)	3830405 (3130737–4579840)	3335478 (2688594–4015344)	494927 (381091–647761)
	ASDR per 10^5^ (95% UI)	438.16 (361.27–527.31)	779.2 (625.79–941.62)	124.49 (94.22–162.31)
	YLDs (95% UI)	85870 (53940–125721)	76024 (47783–111387)	9846 (6063–14791)
	ASYLDR per 10^5^ (95% UI)	9.81 (6.24–14.12)	17.7 (11.26–25.62)	2.4 (1.48–3.57)
	YLLs (95% UI)	3744535 (3066394–4486120)	3259454 (2621956–3939531)	485082 (372122–636439)
	ASYLLR per 10^5^ (95% UI)	428.35 (353.64–514.57)	761.5 (612.38–921.16)	122.09 (92.07–159.03)
**2021**	Deaths (95% UI)	373823 (285552–478421)	331587 (251500–429418)	42236 (27752–60634)
	ASMR per 10^5^ (95% UI)	19.35 (14.79–24.71)	39.9 (29.92–52.05)	4.08 (2.63–5.9)
	DALYs (95% UI)	8593904 (6612569–10883367)	7834073 (5977996–10052681)	759830 (523496–1050934)
	ASDR per 10^5^ (95% UI)	421.02 (326.09–528.39)	820.81 (630.03–1045.28)	70.48 (48.36–98.16)
	YLDs (95% UI)	225512 (140394–321595)	203043 (127224–288389)	22470 (13319–33049)
	ASYLDR per 10^5^ (95% UI)	10.37 (6.5–14.69)	19.28 (12–27.29)	2.01 (1.21–2.96)
	YLLs (95% UI)	8368391 (6392682–10661926)	7631031 (5786889–9859840)	737361 (505820–1030964)
	ASYLLR per 10^5^ (95% UI)	410.65 (315.49–518.61)	801.52 (611.2–1023.37)	68.47 (46.77–96.1)
**EAPC (1990–2021)**	ASMR per 10^5^ (95% CI)	0.64 (0.36–0.91)	1.10 (0.79–1.41)	-1.29 (-1.64 – -0.93)
	ASDR per 10^5^ (95% CI)	0.26 (0.05–0.48)	0.62 (0.39–0.86)	-1.64 (-1.94 – -1.34)
	ASYLDR per 10^5^ (95% CI)	0.51 (0.38–0.65)	0.68 (0.53–0.82)	-0.57 (-0.69 – -0.44)
	ASYLLR per 10^5^ (95% CI)	0.26 (0.04–0.47)	0.62 (0.39–0.86)	-1.67 (-1.97 – -1.36)

ASMR: age-standardized mortality rate. ASDR: age-standardized disability rate. ASYLD: age-standardized years lived with disability. ASYLL: age-standardized years of life lost. DALYs: disability-adjusted life years. YLDs: years lived with disability. YLLs: years of life lost. UI: uncertainty interval. CI: confidence interval. EAPC: estimated annual percentage change.

### Temporal trends in deaths, DALYs, YLDs, and YLLs from smoking-related IHD in China (1990–2021)

**Figure 3:**
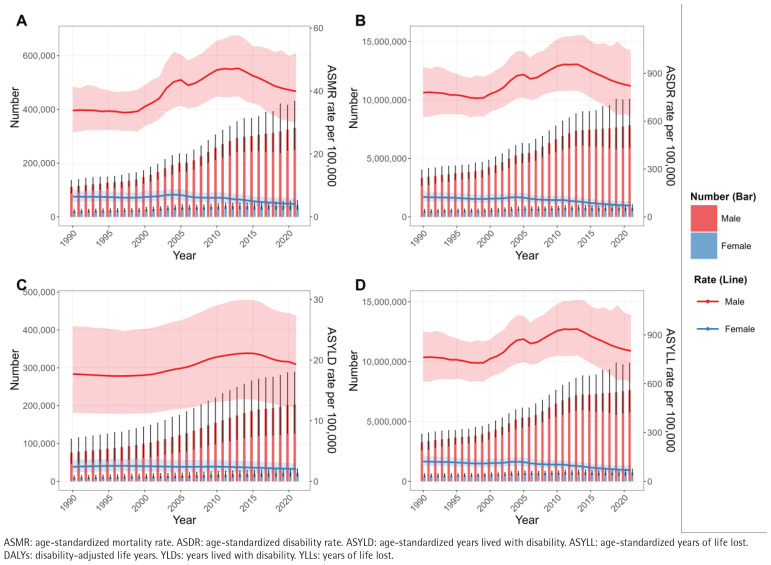
The growing divergence in smoking-related IHD burden between Chinese males and females, 1990–2021: A) Deaths and ASMR, B) DALYs and ASDR, C) YLDs and ASYLD, D) YLLs and ASYLL. Error bars and shaded areas denote 95% uncertainty intervals (UI)

Overall, deaths, DALYs, YLDs, and YLLs have generally increased over the years, with a more significant rise observed in males compared to females. The ASMR, ASDR, ASYLD, and ASYLL metrics for the male population showed a continuous increase from 2000 to 2010, followed by a slight decline after 2010. The estimated annual percentage change (EAPC) from 1990 to 2021 for these metrics in males were 1.10% (95% CI: 0.79–1.41), 0.62% (95% CI: 0.39–0.86), 0.68% (95% CI: 0.53–0.82), and 0.62% (95% CI: 0.39– 0.86), respectively. In contrast, these four indicators for the female population consistently declined from 1990 to 2021, with EAPC values of -1.29% (95% CI: -1.64 –-0.93), -1.64% (95% CI: -1.94 – -1.34), -0.57% (95% CI: -0.69 – -0.44), and -1.67% (95% CI: -1.97 – -1.36), respectively (Figure 3, Table 2).

### Joinpoint regression analysis of ASMR, ASDR, ASYLD, and ASYLL trends in smoking-related IHD in China (1990–2021)

The Joinpoint regression analysis results for the trends in ASMR, ASDR, ASYLD, and ASYLL related to smoking-related IHD in China from 1990 to 2021 reveal distinct patterns. For males, the overall trends for ASMR, ASDR, ASYLD, and ASYLL indicate an upward trajectory, with average annual percentage change (AAPC) values of 0.47 (p<0.05), 0.10 (p=0.48), 0.27 (p<0.05), and 0.10 (p=0.51), respectively. Segmented analysis indicates that during the periods of 1998–2004 and 2007–2010, there were significant increases in all four metrics. However, a marked decline was observed from 2013 to 2021 (p<0.05) (Supplementary file Figure 3).

For females, the overall trends for ASMR, ASDR, ASYLD, and ASYLL demonstrate a downward trajectory, with AAPC values of -1.49 (p<0.05), -1.92 (p<0.05), -0.55 (p<0.05), and -1.95 (p<0.05), respectively. The segmented analysis reveals that, apart from a brief increase in ASMR, ASDR, and ASYLL during the 1998–2004 period, all four metrics have consistently declined over the other time periods (Supplementary file Figure 1).

### Forecasting the burden of smoking-related IHD in China by 2036 using ARIMA model

According to the predictions made by the ARIMA model, the burden of smoking-related IHD in China is expected to be as follows by 2036: ASMR for males is projected to be 39.01 (95% CI: 22.69–55.33) per 100000, while for females it is expected to be 2.76 (95% CI: -0.45, 5.98) per 100000 (Supplementary file Figure 2A); ASDR for males is forecasted at 799.18 (95% CI: 525.68–1072.69) per 100000, and for females it is estimated at 43.94 (95% CI: 9.74–78.13) per 100000 (Supplementary file Figure 2B); ASYLD for males is expected to be 15.53 per 100000 (95% CI: 9.77–21.30 per 100000), while for females it is projected at 0.96 (95% CI: 0.21–1.71) per 100000 (Supplementary file Figure 2C); ASYLL for males is forecasted to be 781.30 (95% CI: 511.65–1050.95) per 100000, and for females it is expected to be 42.20 (95% CI: 8.08–76.31) per 100000 (Supplementary file Figure 2D). Of significance, the ASMR, ASDR, and ASYLL for males are expected to remain stable compared to the levels in 2021, while all indicators for females show a significant decrease.

## DISCUSSION

This study is the first to systematically assess the long-term disease burden trends of smoking-related IHD in China from 1990 to 2021 using GBD and CHARLS data. CHARLS Wave 1 data provide critical population-level evidence linking smoking to cardiovascular health in Chinese adults. While CHARLS recorded heart disease as a broad category without IHD specificity, the robust association observed between smoking status and heart disease likelihood, strongly suggests smoking contributes significantly to the burden of cardiac disease, of which IHD is a major component.

Importantly, the CHARLS analysis revealed a higher likelihood among former smokers compared to never smokers, while the association for current smokers did not reach statistical significance. This apparent discrepancy can be explained by two non-mutually exclusive mechanisms. First, reverse causation is highly plausible: a diagnosis of heart disease often serves as a powerful impetus for smoking cessation. This would lead to a migration of sicker individuals from the current smoker category into the former smoker group, thereby inflating the likelihood estimate for former smokers and concurrently creating a ‘healthy smoker’ bias in the current smoker group who have not developed overt disease. Second, the harmful cardiovascular effects of smoking are cumulative and may manifest most strongly after a period of time, meaning the full likelihood impact is captured in those with a history (former smokers) rather than solely in current users.

The CHARLS analysis results align with our primary findings on smoking-attributable IHD disease burden trends (1990–2021). The ‘lagging-catching up’ pattern of mortality and disability rates for IHD at the national level mirrors the delayed manifestation of smoking-related vascular damage seen in CHARLS, where former smokers’ higher likelihood may reflect cumulative atherosclerotic effects leading to clinical events, including IHD. It reveals a severe situation in which the disease burden of smoking-related ischemic heart disease in China has significantly surpassed international levels. Compared with global and US data, various disease burden indicators in China exhibit a clear ‘lagging-catching up’ characteristic: the ASMR has consistently remained above international levels since 2005, while the ASDR and ASYLL have exceeded the global average since 2011 and 2012, respectively. Furthermore, the ASYLD has been persistently high since 2000. This temporal change pattern may reflect the cumulative impact of multiple factors: first, the acceleration of industrialization in China (2000–2010) led to increased smoking rates and lifestyle changes, resulting in a rapid accumulation of disease burden^17^; second, compared to the well-established cardiovascular disease prevention and control systems in developed countries, deficiencies in early screening and secondary prevention in China may have exacerbated disease progression^18^; third, the accelerated aging process has further amplified the health risks associated with smoking^19^. Importantly, the persistently high ASYLD indicator (reflecting the burden of disability from diseases) suggests significant shortcomings in chronic disease management and rehabilitation services in China.

From the perspective of age distribution, the population aged 50–79 years bears the majority of the absolute disease burden, while the population over 90 years shows the highest age-standardized rate. This ‘bimodal distribution’ phenomenon may reflect two key issues: on the one hand, the high burden in the working-age population indicates significant gaps in the prevention and control of smoking-related cardiovascular risks among occupational groups, especially among men, who often face high work pressure and low health awareness^20-22^; on the other hand, the high standardized rate in the elderly population reflects the severe impact of long-term smoking accumulation on senior health^23^. It is particularly noteworthy that the gender differences identified in the study are significant, with men exceeding women in all indicators, likely related to unique socio-cultural factors in China, including persistently high male smoking rates, differences in health behaviors, and gender imbalances in access to healthcare resources^24,25^. From the composition of disease burden, years of life lost (YLLs) significantly outnumber years lived with disability (YLDs), indicating that smoking primarily leads to premature death rather than long-term disability, which provides important direction for the formulation of prevention and control strategies^12^. These findings strongly advocate for the need to establish targeted intervention measures, particularly early screening and smoking cessation interventions for middle-aged male workers, as well as long-term health management for elderly smokers.

The dynamic evolution of the disease burden of smoking-related IHD in China over the past thirty years shows a significant gender differentiation trend. The male population exhibits a clear ‘rising-slowing’ characteristic, with rapid increases in various indicators from 2000 to 2010, followed by a slight decline after 2010 but still maintaining positive growth. This trajectory aligns closely with key periods of economic development in China and may reflect the combined effects of multiple factors, including persistently high smoking rates among men, increased work pressure, and insufficient early prevention measures during the industrialization process^17^. In contrast, the female population shows a continuous improvement trend, likely related to the long-term low smoking rates among Chinese women and their relatively high health literacy^26^. Importantly, the slight improvement in male indicators after 2010 coincides with the critical implementation of key measures from the Framework Convention on Tobacco Control in China^27^, suggesting that tobacco control policies may have begun to yield positive effects, although the impact remains limited. This trend difference strongly indicates that future prevention and control strategies need to adopt gender-specific interventions, particularly strengthening workplace smoking cessation interventions and cardiovascular health management for male workers, while continuing to improve smoke-free environment construction to consolidate existing achievements.

Through joinpoint regression analysis, three key turning points in the changes in the disease burden of smoking-related IHD in China have been revealed, which hold significant public health implications. The joinpoint-derived AAPC values, which account for these temporal shifts, provide a nuanced complement to the overall EAPC trends, highlighting periods of accelerated increase and subsequent decline that were masked by the linear EAPC model. The male population exhibited a significant increase in disease burden during two periods 1998–2004 and 2007–2010, coinciding with rapid economic growth and social transformation in China^28^. This may reflect the combined effects of several factors: first, the increase in occupational pressure brought about by the reform of state-owned enterprises in 1998 led to a rise in male smoking rates^29^; second, psychological stress during the 2008 global financial crisis may have exacerbated smoking behavior^30^; third, this period coincided with rapid urbanization in China, where behavioral changes may have amplified the harmful effects of smoking^31^. It is worth noting that after 2013, all indicators showed significant declines, and this change coincides with the comprehensive implementation of key measures from the Framework Convention on Tobacco Control and the promotion of the ‘Healthy China’ strategy^27^, indicating that strengthened tobacco control legislation and public health interventions may have begun to show effects. In contrast, the female population, aside from a brief fluctuation during 1998–2004, has shown a continuous improvement trend, which may be related to the long-term low smoking rates among Chinese women and their more proactive health-seeking behaviors^26^. The research results suggest that smoking interventions targeting men, particularly working-age men, need to be further strengthened, while also capitalizing on the improving trend observed since 2013 to continuously enhance the tobacco control policy system and cardiovascular disease prevention network.

The predictive results of this study, based on the ARIMA model, reveal a severe outlook for the future development of the disease burden of smoking-related IHD in China. The predictions indicate that by 2036, the male ASMR will remain at a high level, showing no significant improvement compared to 2021, which presents a significant gap from the goal of reducing premature mortality from major chronic diseases outlined in the ‘Healthy China 2030’ plan^32^. Importantly, the male ASDR and ASYLL also remain at high levels, indicating that the health losses caused by smoking will continue to have a serious impact on the working-age population. This predictive outcome may reflect multiple obstructive factors: first, the enforcement of existing tobacco control policies may be insufficient to offset the increased disease burden brought about by population aging^33^; second, changes in smoking behavior among occupational groups may face deep-rooted social and cultural resistance^34^; third, there is still room for improvement in the coverage and effectiveness of the secondary prevention system for cardiovascular diseases^9^. In contrast, all indicators for women show significant downward trends, further highlighting the necessity of implementing strengthened interventions targeting the male population. The public health significance of the predictive results lies in the fact that if current prevention and control efforts remain unchanged, it may be difficult for China to achieve a substantial reduction in the disease burden related to smoking. There is a critical need to adopt stricter tobacco control measures. Additionally, the relatively large confidence intervals in the predictions suggest significant uncertainty in the future development of the disease burden, providing potential space for policy adjustments and innovative interventions.

### Limitations

The study has some limitations. First, the estimation nature of GBD data may affect accuracy. Second, the impact of new tobacco products has not been analyzed. Third, given the cross-sectional design of CHARLS, causal relationships cannot be established. While the CHARLS data provided valuable evidence on the smoking-heart disease association, the lack of IHD specificity in its outcome variable is a limitation. However, this broad definition captures the overall cardiovascular risk landscape, and the strong concordance with the GBD’s IHD-specific trends strengthens our conclusions. Future longitudinal studies incorporating specific IHD diagnoses within cohorts like CHARLS would be invaluable for more granular analyses. Fourth, ARIMA forecasting relies on the stability of historical data and does not capture uncertainties arising from dynamic changes in smoking behaviors or policy environments, which may affect the accuracy of long-term projections; particularly, the model does not account for potential disruptions from emerging tobacco products like e-cigarettes, which could alter smoking prevalence and disease burden trajectories, thus limiting the robustness of forecasts. Fifth, the population-level nature of the data may introduce ecologic associations, limiting individual-level inferences. Additionally, residual confounding may persist due to unmeasured factors such as socioeconomic status or dietary habits. Multiple comparisons in the analysis could inflate Type I error. Finally, the generalizability of the findings may be limited to the Chinese context, as the study relies on data from a specific population, and results might not be directly applicable to other regions with different demographic or cultural characteristics.

## CONCLUSIONS

This study systematically outlines the disease burden profile of smoking-related IHD in China, revealing that the burden exceeds global averages with significant gender disparities – males bear a disproportionately higher burden, while females show improving trends. These insights underscore the necessity for further research to explore regional variations, the impact of emerging tobacco products, and longitudinal studies to establish causal relationships and inform more precise public health interventions.

## Supplementary Material



## Data Availability

The data supporting this research are available from the authors on reasonable request.
