# Supplementary material for: Smoking-attributable ischemic heart disease burden in China from 1990 to 2021 and projections to 2036: A retrospective analysis and forecasting study
Source: Tob Induc Dis. 2026 Mar 21;24:10.18332/tid/217625. doi: 10.18332/tid/217625 (PMC13005603; doi:10.18332/tid/217625)
Supplement: Supplementary file 1 [file TID-24-41-s1.pdf]

Supplementary File

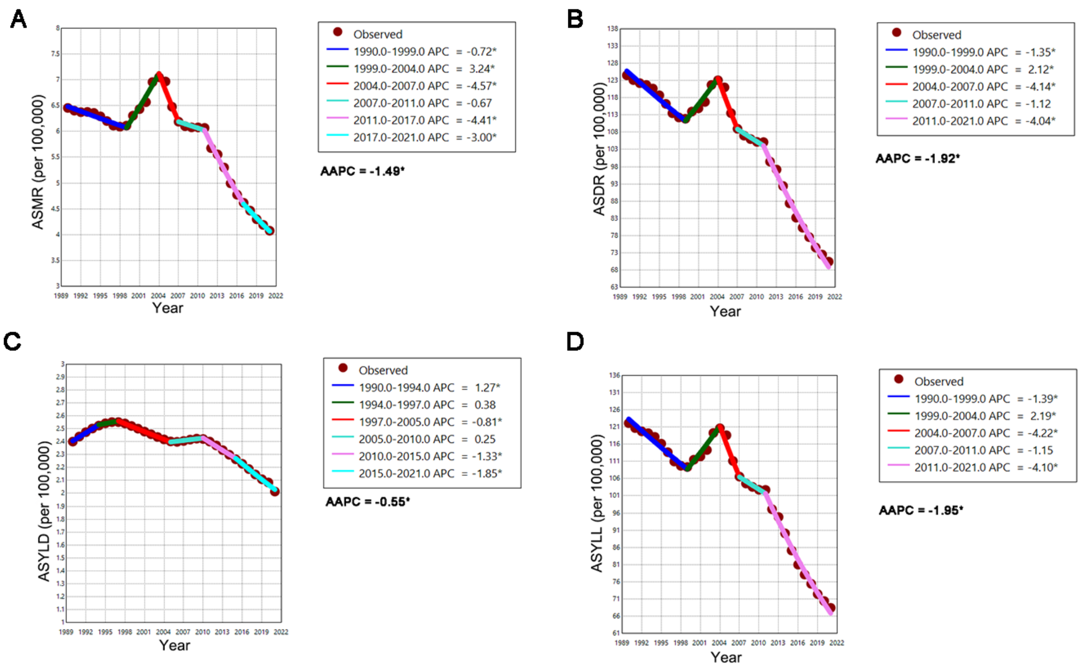

Supplementary Figure 1. Joinpoint Regression Analysis of ASMR (A), ASDR (B), ASYLD (C), and ASYLL (D) Trends in Female Smoking-Related Ischemic Heart Disease in China (1990-2021). * Indicates that the APC is significantly different from zero at the alpha = 0.05 level. Notes: ASMR, Age-Standardized Mortality Rate; ASDR, Age-Standardized Disability Rate; ASYLD, Age-Standardized Years Lived with Disability; ASYLL, Age-Standardized Years of Life Lost; APC, Annual Percentage Change; AAPC, Average Annual Percentage Change.

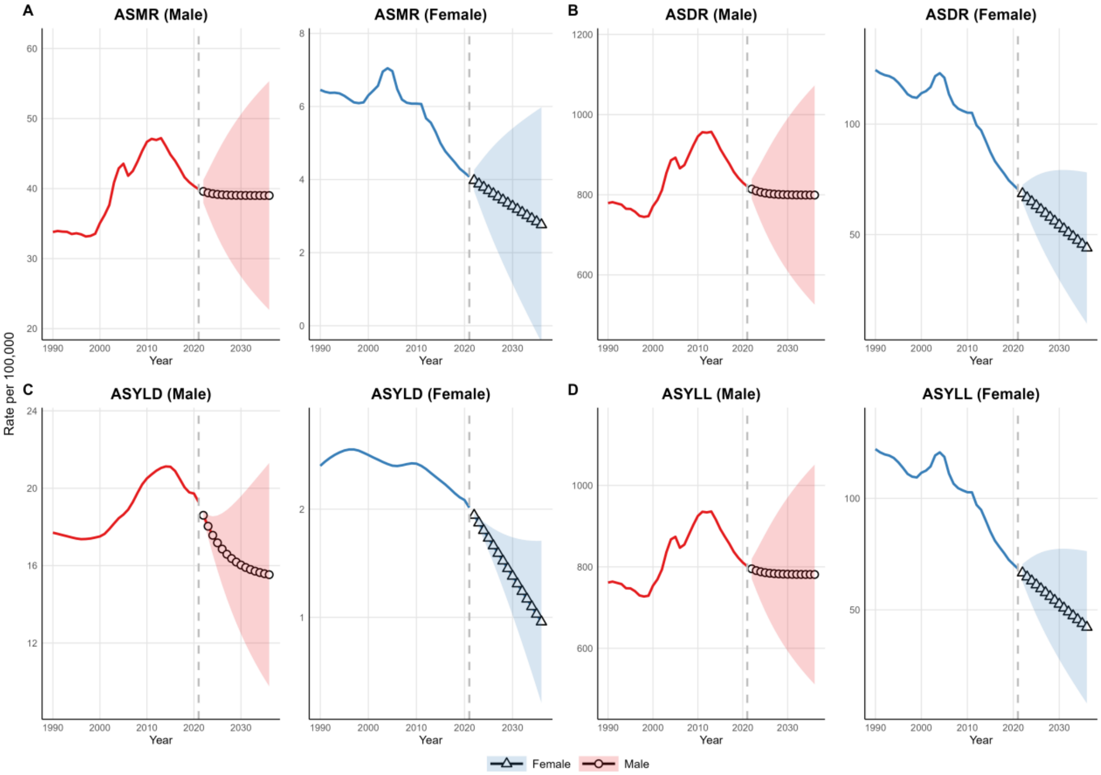

Supplementary Figure 2. Forecasting the Burden of Smoking-Related IHD in China by 2036 Using ARIMA Model. (A) ASMR. (B) ASDR. (C) ASYLD. (D) ASYLL. The shaded areas show 95% confidence intervals for future burden estimates. Notes: ASMR, Age-Standardized Mortality Rate; ASDR, Age-Standardized Disability Rate; ASYLD, Age-Standardized Years Lived with Disability; ASYLL, Age-Standardized Years of Life Lost.

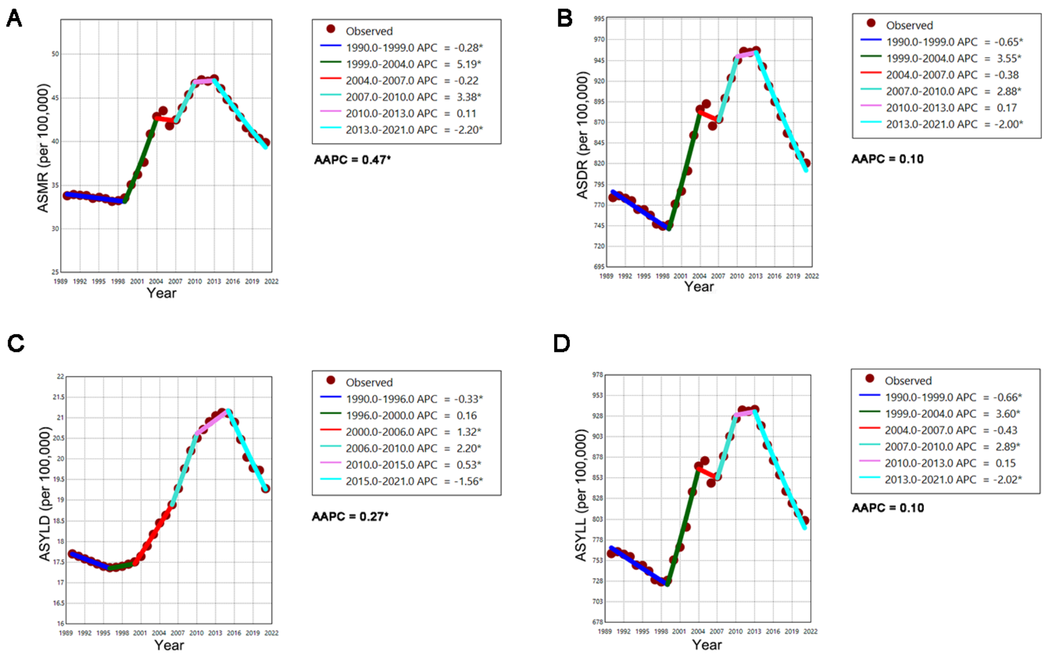

Supplementary Figure 3. Joinpoint regression analysis of ASMR (A), ASDR (B), ASYLD (C), and ASYLL (D) trends in male smoking-related IHD in China (1990–2021). Notes: *APC is significantly different from zero at the alpha = 0.05 level. ASMR: age-standardized mortality rate. ASDR: age-standardized disability rate. ASYLD: age-standardized years lived with disability. ASYLL: age-standardized years of life lost. APC: annual percentage change. AAPC: average annual percentage change.

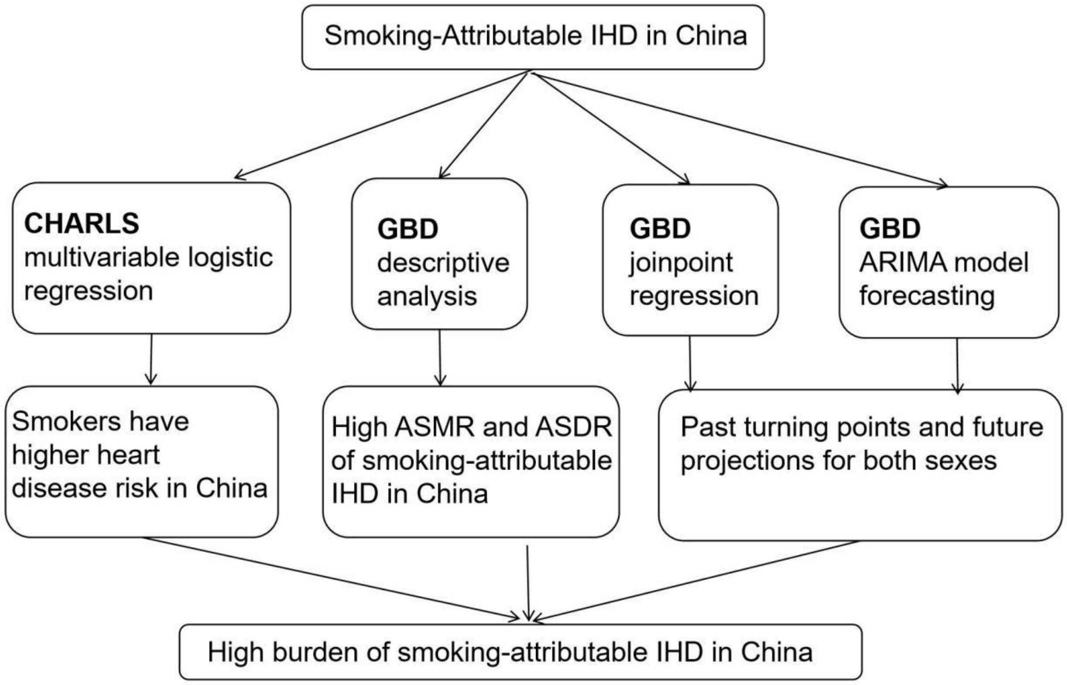

Supplementary Figure 4. The Heavy and Disparate Burden of Smoking-Attributable Ischemic Heart Disease in China: Past Trends and Future Projections.

© 2026 Xu D. et al.
